# Context-dependence of race self-classification: Results from a highly mixed and unequal middle-income country

**DOI:** 10.1371/journal.pone.0216653

**Published:** 2019-05-16

**Authors:** Dóra Chor, Alexandre Pereira, Antonio G. Pacheco, Ricardo V. Santos, Maria J. M. Fonseca, Maria I. Schmidt, Bruce B. Duncan, Sandhi M. Barreto, Estela M. L. Aquino, José G. Mill, Maria delCB Molina, Luana Giatti, Maria daCC Almeida, Isabela Bensenor, Paulo A. Lotufo

**Affiliations:** 1 Department of Epidemiology and Quantitative Methods, National School of Public Health, Oswaldo Cruz Foundation, Rio de Janeiro, RJ, Brazil; 2 Laboratory of Genetics and Molecular Cardiology, Heart Institute (InCor), University of São Paulo, São Paulo, SP, Brazil; 3 Scientific Computing Program, Oswaldo Cruz Foundation, Rio de Janeiro, RJ Brazil; 4 Department of Anthropology, Museu Nacional, Rio de Janeiro, RJ Brazil; 5 Postgraduate Program in Epidemiology, School of Medicine, Federal University of Rio Grande do Sul, Porto Alegre, RS Brazil; 6 Faculty of Medicine & Clinical Hospital, Federal University of Minas Gerais, Belo Horizonte, MG Brazil; 7 Institute of Collective Health, Federal University of Bahia, Salvador, BA Brazil; 8 Department of Physiological Sciences, Federal University of Espirito Santo, Vitória, ES Brazil; 9 Gonçalo Muniz Institute, Oswaldo Cruz Foundation, Salvador, BA Brazil; 10 Center for Clinical and Epidemiological Research, University Hospital, University of São Paulo, São Paulo, SP Brazil; University of California San Francisco, UNITED STATES

## Abstract

Ethnic-racial classification criteria are widely recognized to vary according to historical, cultural and political contexts. In Brazil, the strong influence of individual socio-economic factors on race/colour self-classification is well known. With the expansion of genomic technologies, the use of genomic ancestry has been suggested as a substitute for classification procedures such as self-declaring race, as if they represented the same concept. We investigated the association between genomic ancestry, the racial composition of census tracts and individual socioeconomic factors and self-declared race/colour in a cohort of 15,105 Brazilians. Results show that the probability of self-declaring as black or brown increases according to the proportion of African ancestry and varies widely among cities. In Porto Alegre, where most of the population is white, with every 10% increase in the proportion of African ancestry, the odds of self-declaring as black increased 14 times (95%CI 6.08–32.81). In Salvador, where most of the population is black or brown, that increase was of 3.98 times (95%CI 2.96–5.35). The racial composition of the area of residence was also associated with the probability of self-declaring as black or brown. Every 10% increase in the proportion of black and brown inhabitants in the residential census tract increased the odds of self-declaring as black by 1.33 times (95%CI 1.24–1.42). Ancestry alone does not explain self-declared race/colour. An emphasis on multiple situational contexts (both individual and collective) provides a more comprehensive framework for the study of the predictors of self-declared race/colour, a highly relevant construct in many different scenarios, such as public policy, sociology and medicine.

## Introduction

While there is a long debate in science whether the biological concept of race is applicable to the human species [1–3], it is widely recognized that race as a social construct, with all its consequences, deeply influences daily life. Often included as a variable in social and biomedical research, as well as central to a broad range of public policies, race classification varies throughout the world. Influenced by historic, economic, and cultural factors, race classification systems are associated with several consequences, ranging from the likelihood of accessing different educational opportunities, professional status, neighbourhood of residence, to current medical practice of labelling patients with increased or decreased risk of developing specific medical conditions.

The complex historical formation of the Brazilian population, marked by admixture and resulting from the interaction between European, African and Amerindian populations, has led to Brazil being one of the countries where racial classification has been most intensely investigated over the past decades [4–11]. There was a time when racial relations in Brazil were believed to be less tense than in other countries, what was described as a “racial democracy”. As a result of significant anthropological and sociological research efforts carried out since the 1950s, in addition to work by black activists, not only has racism in Brazil been widely acknowledged, but so has the fluidity of racial categorizations, which are influenced by socio-economic attributes, in contrast to other parts of the world, where self-classification patterns are less permeated by cultural and socio-economic contexts [6,9,12–14].

In recent years, as a result of the development and dissemination of gene technologies, the investigation of genomic profiles to characterize biogeographical ancestry has become increasingly common. In Brazilian health research, there is a clear interest in evaluating the levels of agreement between ethno-racial profiles derived from the analysis of genomic markers *vis-a-vis* those deriving from traditional classification procedures, especially self-classification [15–23]. Though not always explicitly stated, a line of reasoning found in some of the most recent investigations pertains to the advantages and limitations of using genomic ancestry as a complement to, or even in substitution of (as if they represented the same concept), more commonly used methodological procedures, such as self-declared race or colour [24]. In this line of reasoning, Mola et al. (2016) [24], in a study that sought to investigate relationships between depression, self-perceived race or colour and genomic ancestry, wrote: “In highly genetically admixed populations, such as in Brazil, personal information on ethnicity might not provide the same robust estimations as in less diverse populations… [S]elf-perception of skin race/colour could be biased by the context in which a person live [sic], especially in Brazil, where different regions of the country present different levels of admixture…It is not our objective to establish if certain specific genetic variations, used to assess ancestry, are associated with depression, but to use them as a whole, as a proxy of skin race/colour” (2016:2).

Since race or colour has been used in different social situations and by different scientific communities, and is deeply associated with different measures of social conditions, it is important to develop a framework to better understand the factors that predict self-declared race or colour, including biological ancestry. This is even more important in populations in which both admixture and socio-economic and racial inequalities are high, since different societal, economic and cultural pressures may be affecting the inter-relationships between those factors in different and maybe unpredictable ways. As such, by simultaneously identifying to what extent race classifications are dependent on context and genomic ancestry, we may develop a better understanding of the different factors at play. This may be used to predict, identify and act upon potential scenarios that may be working as positive feedback loops, leading to unequal and discriminatory allocation of resources.

In this article, we have used data from one of the most comprehensive ongoing cohort studies in Brazil to investigate the association between self-declared race/colour and a range of social, contextual (census tracts) and genetic factors. We show, based on the use of data from 9,333 genotyped participants from six different cities in Brazil, that emphasis on multiple situational contexts (both individual and collective) provides a more complete framework for the study of the predictors of self-declared race/colour, a highly relevant construct in several different scenarios, such as public policy, sociology, economics and medicine.

## Materials and methods

### Participants

The Brazilian Longitudinal Study of Adult Health (ELSA-Brasil) is a multi-centre prospective cohort study designed primarily to identify risk factors and the natural history of diabetes and cardiovascular disease (CVD) [25,26]. The cohort comprises 15,105 active or retired employees of universities or research institutions located in six Brazilian capitals (Salvador, Vitória, Belo Horizonte, Rio de Janeiro, São Paulo, Porto Alegre) who were 35–74 years of age at baseline (2008–2010). ELSA-Brasil was performed in accordance with relevant guidelines and regulations, and all participants from ELSA-Brasil gave written informed consent to participate in the study. ELSA-Brasil was approved by the Ethics Committees of the Hospital das Clínicas de Porto Alegre, Hospital Universitário da Universidade de São Paulo, Fundação Oswaldo Cruz, Universidade Federal de Minas Gerais, Universidade Federal da Bahia, and Universidade Federal do Espírito Santo.

Among the 15,105 ELSA-Brasil participants, 9,834 (65%) were genotyped. Proportions varied between 25% in Porto Alegre and 91% in São Paulo. Of these, 116 (1,2%) did not have valid values for race/colour and were therefore excluded from the analysis. Among participants who self-declared as white, 65% were genotyped. Among participants who self-declared as black, brown, of Asian descent (mostly individuals with East Asian ancestors) and indigenous, this proportion was 62%, 65%, 75% and 66%, respectively.

The comparison between genotyped participants, who were included in this study, and non-genotyped participants can be found in the S1 Table. Given the study objectives and the small number of participants who self-declared as being of Asian descent (N = 281) or indigenous (104), these two groups were also excluded. Thus, the final sample included 9,333 participants. When compared with excluded participants, the study population had a higher proportion of white and brown participants and lower educational and income levels.

Questionnaires: The main predictor variables were sex, age in years (continuous), self-declared skin race/colour (white, black, brown, indigenous and of Asian descent; “brancos”, “pretos”, “pardos”, “indígenas” and “amarelos”), formal educational level achieved (until secondary education or university), per capita income and current residential address.

### Geocoding

Neighbourhood environments were based on study-defined boundaries which were created for each site using a spatial aggregation method based on SKATER (Spatial ‘K’luster Analysis by Tree Edge Removal). This method was used to create clusters of contiguous census tracts that had a minimum population size of 5,000 inhabitants and were homogeneous with regard to four socio-economic indicators from the 2010 Brazilian Institute of Geography and Statistics (IBGE) demographic census [27]: the proportion of the population 0 to 4 years of age; household size; mean household income; and the proportion of white inhabitants [28]. This geographic scale is comparable in size to U.S. census tracts which are commonly-used proxies in neighbourhood effects research [29].

### Global genetic ancestry determination

Purified DNA was obtained from peripheral blood from ELSA’s participants using the QIAamp DNA Mini-kit®. Samples were genotyped using a 192 AIM panel shown to be able to capture main continental ancestry components in the Brazilian population [30]. Genotyping was performed with the QuantstudioTM platform. Briefly, a multiplex TaqMan reaction was conducted in each sample for the 192 SNP panel according to manufacturer instructions. Each genotyping run carried 2 control genotypes (samples sequenced for all 192 variant alleles) and one negative control. Fluorescent results were analyzed in the QuantStudioTM and genotyped using the TaqMan_Genotyper, version 1.3 (Life Technologies, Foster City, CA, EUA) using the ‘autocalling’ tool for genotype assignment. Genotyping experiments were considered valid only with a call rate above 80% and with 100% of concordance for genotyping assignment for control samples.

Analysis of genomic ancestry was conducted using the ADMIXTURE program [31]. ADMIXTURE is a software tool for maximum likelihood estimation of individual ancestries from multilocus SNP genotype datasets. Specifically, Admixture uses a block relaxation approach to alternately update allele frequency and ancestry fraction. ADMIXTURE estimates parameter standard errors using bootstrapping. As the contributions of differential ancestral genomes have previously been described by our group, as well as others, we used a supervised approach for ancestry determination. We used 200 bootstrap replicates (default) and k = 3 (number of populations assumed for the analysis). All ADMIXTURE analyses were repeated 4 times with different random seed numbers and in all cases, results were highly correlated.

We assumed as reference ancestral populations individuals from the Human Genome Diversity Project (HGDP): Pima, Maya as Amerindians and from the HapMap project, Africans: YRI (Yoruba in Ibadan, Nigeria), LWK (Luhya in Webuye, Kenya), ASW (Americans of African Ancestry in SW, USA); European: CEU (Utah Residents with Northern and Western European ancestry) and TSI (Tuscan in Italy). Ancestry variables are analysed as ancestry fractions (continuous).

### Statistical analysis

Descriptive statistics included the median and interquartile range for continuous variables and frequency distributions for categorical variables. Comparisons were tested with the nonparametric Mann–Whitney test for continuous variables (for 2 groups) and Kruskal-Wallis (for 3 or more groups) and Chi-squared tests for categorical variables. Boxplots were used to describe the proportional distribution of ancestry per self-classification categories. In order to identify the most adequate cut-off point for the proportion of black and brown inhabitants in the census tracts (≥60%), we inspected the graphs showing the predicted probabilities of self-declaring as white, brown or black according to the percentage of black and brown inhabitants in the census tract. We used the multinomial model with smoothing splines. We found inflection points on the curves and the 60% cut-off seemed like a good inflection point for white and brown individuals, less evident for black individuals. Logistic regression models with mixed effects were used to calculate odds ratios (ORs) between exposure variables and the main outcome, ethnoracial self-classification. Probability curves of self-classification over ancestry proportions were predicted with multinomial logistic models. We tested the interaction between ancestry-education, ancestry-income and ancestry-proportion of blacks and browns in the census tracts of residence.

## Results

### Participants’ characteristics

Most participants in this study have high educational levels (50.8% had a university degree) and self-declared as white (54.6%), whereas 29.4% and 16% self-declared as brown and black, respectively (Table 1). The study population also consisted of younger individuals and marginally more women (53.8%). Only in Salvador did most participants self-declare as brown or black. Median income was US$ 362 (monthly per capita income in United States dollars (USD), conversion rate: 2 Brazilian reais  =  1USD). Median proportion of African, European and Amerindian ancestry was 20%, 70% and 10%, respectively. Only participants in Salvador presented a higher proportion (40%) of African ancestry and only those in Porto Alegre presented a higher proportion (80%) of European ancestry. In the census tracts where participants reside, 32.9% of inhabitants self-declared as brown or black. This proportion varied between 75.1% in Salvador and 11.9% in Porto Alegre.

**Table 1:** Selected characteristics by research centre and total. ELSA-Brasil, 2008–2010.

Variables	Salvador	Vitória	Belo Horizonte	Rio de Janeiro	São Paulo	Porto Alegre	Overall	P-value
**Total**	938	556	1473	1574	4289	503	9333	-
**Color/Race, N(%)**								
White	180 (19.2)	249 (44.8)	731 (49.6)	871 (55.3)	2691 (62.7)	370 (73.6)	5092 (54.6)	<0.001*
Brown	424 (45.2)	250 (45.0)	561 (38.1)	489 (31.1)	971 (22.6)	51 (10.1)	2746 (29.4)	
Black	334 (35.6)	57 (10.3)	181 (12.3)	214 (13.6)	627 (14.6)	82 (16.3)	1495 (16.0)	
**Educational level, N(%)**								
Completed secondary	513 (54.7)	244 (43.9)	639 (43.4)	478 (30.4)	2446 (57.0)	273 (54.3)	4593 (49.2)	<0.001*
University or +	425 (45.3)	312 (56.1)	834 (56.6)	1096 (69.6)	1843 (43.0)	230 (45.7)	4740 (50.8)	
**Sex, N(%)**								
M	431 (45.9)	274 (49.3)	667 (45.3)	754 (47.9)	1983 (46.2)	200 (39.8)	4309 (46.2)	0.025*
F	507 (54.1)	282 (50.7)	806 (54.7)	820 (52.1)	2306 (53.8)	303 (60.2)	5024 (53.8)	
**Income (USD), N(%)**								
<500.00	435 (46.5)	221 (39.8)	541 (36.8)	313 (19.9)	1833 (42.9)	189 (37.7)	3532 (38.0)	<0.001*
501.00 to 1000.00	292 (31.2)	201 (36.2)	531 (36.1)	668 (42.5)	1358 (31.8)	171 (34.1)	3221 (34.6)	
>1000.00	208 (22.2)	133 (24.0)	398 (27.1)	591 (37.6)	1079 (25.3)	141 (28.1)	2550 (27.4)	
**Age**								
median (IQR)	54 (47–61)	53 (46.8–60)	51 (45–57)	49 (44–54)	50 (45–57)	50 (45–57)	50 (45–57)	<0.001**
**African ancestry proportion**								
median (IQR)	38 (17.5–60.5)	13.5 (4.1–27.5)	16.1 (7.5–31.1)	16 (6.6–30.3)	17.5 (6–35.1)	6.9 (1.4–16.5)	17.2 (6.4–35.4)	<0.001**
**European ancestry proportion**								
median (IQR)	53.1 (31.4–73.6)	74.6 (56–87.5)	75.8 (60.2–85.7)	69.4 (47–83.2)	67.6 (37.2–84.6)	82.2 (61.5–90.4)	69.7 (43.8–84.6)	<0.001**
**Amerindian ancestry proportion**								
median (IQR)	6.9 (3.8–10.6)	9.5 (5.7–14.9)	6.7 (3.7–9.9)	12.1 (7.5–18.8)	12 (6.5–20.4)	9.9 (5.3–16.4)	9.9 (5.5–16.5)	<0.001**
Brown/Black proportion***								
median (IQR)	75.1 (56.9–85.3)	41.7 (29.4–60.6)	42.8 (25.7–59.1)	31.2 (16.6–49.8)	24.3 (11.7–41.7)	11.9 (5.8–24.8)	32.9 (16–52.5)	<0.001**

*Chisq test

**Kruskal-Wallis test

***by census tracts

**Fig 1:**
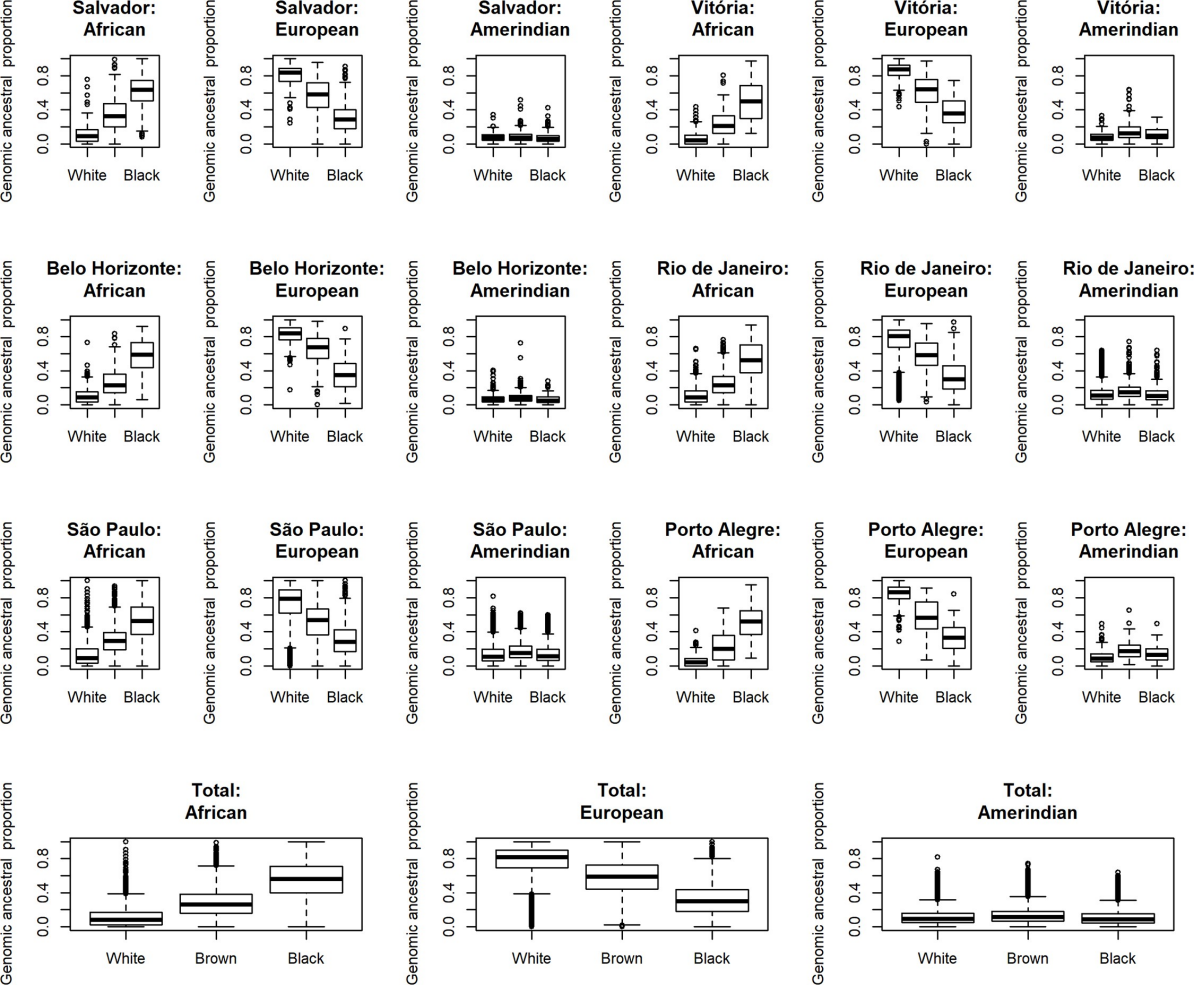
Box plot contrasting ethnoracial self-classification (white, brown and black) to median individual proportion of genomic African, European and Amerindian ancestries in all participants, and by research centre (ELSA-Brasil).

In ELSA-Brasil, among those who self-declared as black or brown, the proportion of African ancestry was 56.4% and 26.5%, respectively (Fig 1). Among those who self-declared as brown, the proportion of African ancestry varied between 20% in Porto Alegre and 32.8% in Salvador. Among this group, the proportion of European ancestry was 58.9%, varying between 58.1% in Salvador and 67.9% in Belo Horizonte. Medians of African, European and Amerindian proportions according to research centres and self-declared race/colour can be found in S2 Table.

### Odds of self-declared race/colour

Table 2 shows the age, sex, education, income and African ancestry adjusted odds ratios of self-declaring as black, when compared to white, according to selected characteristics (S3 Table shows those results using the proportion of black inhabitants in the census tracts, instead of black and brown inhabitants). Depending on the proportion of black and brown inhabitants in the census tracts (we observed an interaction between genomic ancestry and the racial composition of the census tracts), a 10% increase in African ancestry increases the odds of self-declaring as black by 3.42 (3.16–3.70) times and by 4.09 (3.30–5.05) in census tracts with less than 60% and with 60% or more blacks and browns, respectively. Having high educational level–university degree–reduced the odds of self-declaring as black by 63% (OR = 0.37; 0.28–0.49) in census tracts with less than 60% whereas the reduction was not statistically significant in those with 60% or more blacks and browns (OR = 0.59; 0.29–1.19). As in the case of schooling, having high per capita income levels (over US$1,000.00) reduced significantly the odds of self-declaring as black in areas with a lower proportion of blacks and browns (OR = 0.51; 0.36–0.72) but it was not statistically significant in areas with 60% or more blacks and browns (OR = 0.60; 0.25–1.47). Women were 22% more likely than men to self-declare as black only in census tracts with less than 60% of blacks and whites. The effect of age was not important except in Salvador, where each 10-year increase of age decreased the odds of self-declaring as black by 39% (OR = 0.61; 0.38–0.97). Regarding the interaction between ancestry and racial composition of census tracts, we did not identify a specific pattern in the research centres analysed separately.

**Table 2:** Odds ratios of self-declaring as black (compared to white) according to research centre and total. ELSA-Brasil, 2008–2010.

	Research Center						Overall, stratified by Black/Brown proportion in the census tracts	
Variables	Salvador (n = 502)	Vitória (n = 304)	Belo Horizonte (n = 908)	Rio de Janeiro (n = 1080)	São Paulo (n = 3298)	Porto Alegre (n = 445)	Proportion<60% (n = 5732)	Proportion > = 60% (n = 805)
	OR# (95% CI)	OR# (95% CI)	OR# (95% CI)	OR# (95% CI)	OR# (95% CI)	OR# (95% CI)	OR# (95% CI)	OR# (95% CI)
African ancestry (10%)*	3.98 (2.96–5.35)	9.31 (4.11–21.13)	8.98 (5.73–14.07)	4.41 (3.5–5.57)	2.84 (2.61–3.1)	14.12 (6.08–32.81)	3.42 (3.16–3.70)	4.09 (3.30–5.05)
**Educational level**								
(Ref = completed secondary)	1.00	1.00	1.00	1.00	1.00	1.00	1.00	1.00
University or +	0.27 (0.1–0.7)	1.46 (0.28–7.54)	0.42 (0.16–1.07)	0.73 (0.36–1.48)	0.48 (0.33–0.68)	1.12 (0.13–9.72)	0.37 (0.28–0.49)	0.59 (0.29–1.19)
**Income (USD)**								
(Ref = <500.00)	1.00	1.00	1.00	1.00	1.00	1.00	1.00	1.00
501.00 to 1000.00	0.46 (0.16–1.31)	0.7 (0.15–3.26)	1.62 (0.59–4.5)	0.18 (0.08–0.41)	0.66 (0.48–0.92)	0.49 (0.07–3.46)	0.52 (0.39–0.69)	0.69 (0.33–1.44)
>1000.00	0.95 (0.34–2.64)	0.28 (0.04–2.25)	1.49 (0.41–5.4)	0.2 (0.08–0.48)	0.64 (0.4–1.01)	1.03 (0.1–10.66)	0.51 (0.36–0.72)	0.60 (0.25–1.47)
Age (10 years)**	0.61 (0.38–0.97)	1.12 (0.49–2.56)	0.87 (0.55–1.38)	1.09 (0.76–1.56)	1.08 (0.91–1.29)	1.08 (0.46–2.52)	0.96 (0.83–1.11)	1.00 (0.72–1.39)
**Sex**								
(Ref = Male)	1.00	1.00	1.00	1.00	1.00	1.00	1.00	1.00
Female	0.94 (0.42–2.12)	0.92 (0.26–3.25)	0.77 (0.33–1.77)	2.66 (1.26–3.98)	1.17 (0.88–1.53)	1.11 (0.23–5.24)	1.22 (0.97–1.54)	1.08 (0.62–1.89)
Brown/Black proportion (10%)***	1.13 (0.88–1.47)	1.44 (0.92–2.25)	1.07 (0.83–1.39)	1.31 (1.11–1.55)	1.36 (1.25–1.49)	1.32 (0.65–2.68)	-	-

# mutually adjusted

* results for increases of 10% of African ancestry

** results for increases of 10 years of age

***by census tracts

The odds of self-declaring as black, when compared to white, strongly varied according to geographical location and, accordingly, the interaction term between race/colour and research centre was significant (p<0.001) (Table 2). In Porto Alegre, where most of the population is white, with every 10% increase in the proportion of African ancestry, the odds of self-declaring as black increased 14 times (6.08–32.81). In Salvador, where most of the population is black or brown, that increase was of 3.98 (2.96–5.35) times, while in São Paulo, where the population is more evenly distributed among these racial groups, it was 2.84 (2.61–3.10) times. On the other hand, the proportion of black and brown inhabitants in the census tracts only significantly increased the odds of self-declaring as black in Rio de Janeiro and São Paulo, by 31% (1.11–1.55) and 36% (1.25–1.49), respectively.

Table 3 shows the age, sex, education, income and African ancestry adjusted odds ratios of self-declaring as brown when compared to white, for the entire study population and for each research centre. A 10% increase in the proportion of African ancestry doubled (OR = 2.06;1.97–2.16) the odds of self-declaring as brown in census tracts with less than 60% blacks and browns and increased the same odds by 2.51 (2.17–2.9) in areas with 60% or more black and brown inhabitants. When compared to the lower educational level, having a university degree decreased the odds of self-declaring as brown by 54% (OR = 0.46;0.40–0.54) and by 42% (OR = 0.58; 0.38–0.87) in areas with less than 60% and with 60% or more blacks and browns, respectively. Having high income levels significantly decreased the odds of self-declaring as brown in the two strata of different racial composition. Each 10-year increase of age decreased the odds of self-declaring as brown in the two strata of racial composition and especially in Salvador (OR = 0.66; 0.52–0.84). As for variations between research centres, in Porto Alegre, a 10% increase in African ancestry increased the odds of self-declaring as brown by about four times (2.86–6.43), while in São Paulo that increase was of 1.81 (1.71–1.92) times. A 10% increase in the proportion of black and brown inhabitants in the census tract significantly influenced self-declaring as brown in all the cities except Salvador. A 10% increase in the proportion of black and brown inhabitants in the census tract significantly influenced self-declaring as brown in Vitoria, São Paulo, Belo Horizonte and Rio de Janeiro.

**Table 3:** Odds ratios of self-declaring as brown (compared to white) according to research centre. ELSA-Brasil, 2008–2010.

	Research Center						Overall, stratified by Black/Brown proportion in the census tracts	
Variables	Salvador (n = 588)	Vitória (n = 494)	Belo Horizonte (n = 1290)	Rio de Janeiro (n = 1350)	São Paulo (n = 3640)	Porto Alegre (n = 413)	Proportion<60% (n = 6804)	Proportion> = 60% (n = 971)
	OR# (95% CI)	OR# (95% CI)	OR# (95% CI)	OR# (95% CI)	OR# (95% CI)	OR# (95% CI)	OR# (CI 95%)	OR# (CI 95%)
African ancestry (10%)*	2.74 (2.22–3,37)	3.68 (2.83–4,79)	2.75 (2.39–3.17)	2.04 (1.83–2.27)	1.81 (1.71–1.92)	4.29 (2.86–6.43)	2.06 (1.96–2.16)	2.51 (2.17–2.9)
**Educational level**								
(Ref = completed secondary)	1.00	1.00	1.00	1.00	1.00	1.00	1.00	1.00
University or +	0.55 (0.3–1.01)	0.95 (0.52–1.74)	0.82 (0.59–1.15)	0.49 (0.34–0.69)	0.49 (0.39–0.61)	4.14 (1.31–13.02)	0.46 (0.40–0.54)	0.58 (0.38–0.87)
**Income (USD)**								
(Ref = <500.00)	1.00	1.00	1.00	1.00	1.00	1.00	1.00	1.00
501.00 to 1000.00	1.01 (0.55–1.88)	1.0 (0.55–1.83)	0.76 (0.54–1.08)	0.64 (0.43–0.94)	0.7 (0.57–0.87)	0.3 (0.1–0.86)	0.57 (0.49–0.67)	1.14 (0.76–1.71)
>1000.00	0.79 (0.41–1.53)	0.42 (0.2–0.85)	0.71 (0.47–1.06)	0.6 (0.39–0.91)	0.43 (0.31–0.58)	0.14 (0.04–0.55)	0.43 (0.35–0.52)	0.57 (0.34–0.97)
Age (10 years)**	0.66 (0.52–0.84)	0.92 (0.7–1.2)	0.91 (0.78–1.07)	0.99 (0.83–1.17)	0.95 (0.85–1.06)	0.87 (0.54–1.4)	0.89 (0.82–0.96)	0.86 (0.7- .05)
**Sex**								
(Ref = Male)	1.00	1.00	1.00	1.00	1.00	1.00	1.00	1.00
Female	1.15 (0.74–1.8)	0.79 (0.5–1,27)	0.95 (0.72–1.24)	0.93 (0.71–1.21)	0.85 (0.71–1.01)	1.21 (0.55–2.69)	0.89 (0.79–1.01)	0.81 (0.58–1.14)
Brown/Black proportion (10%)***	1.06 (0.91–1.22)	1.17 (1.0–1.36)	1.1 (1.1–1.2)	1.18 (1.08–1.28)	1.3 (1.23–1.38)	1.3 (0.9–1.88)	-	-

# mutually adjusted

*results for increases of 10% of African ancestry

** results for increases of 10 years of age

***by census tracts

### Predicted probabilities of self-declared race/colour according to genomic ancestry

**Fig 2:**
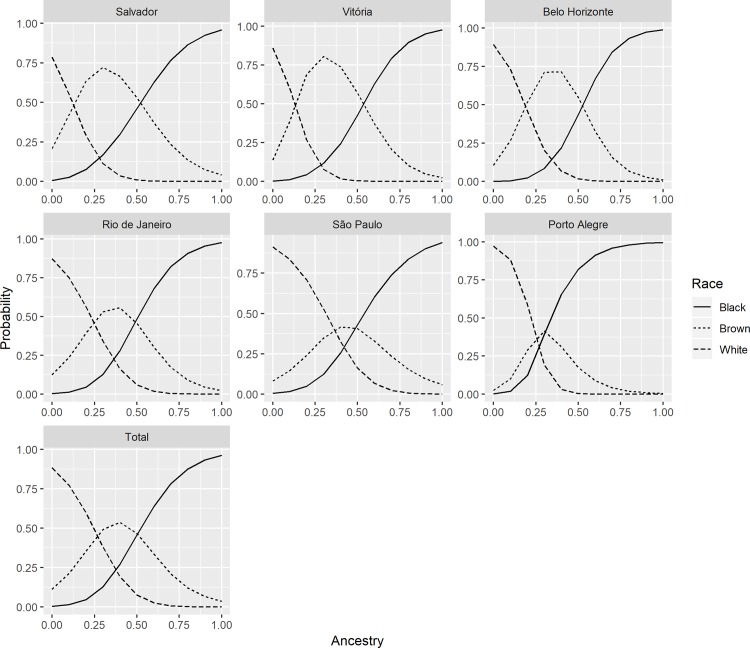
Predicted probability of ethnoracial self-classification as black, brown and white along the continuum of genomic proportion of African ancestry in all participants, and by research centre (ELSA-Brasil).

The joint analysis and the analysis by research centre population of the predicted probabilities of self-declaring as black, brown and white along the African ancestry continuum is shown in Fig 2. African genomic ancestry showed an S-shaped positive correlation with self-reporting as black, which was consistent in all populations, whereas the reverse was observed for self-reporting as white. In the joint analysis, as well as for each research centre separately, these trends were statistically significant (p<0.001 in likelihood ratio tests). The probability of self-reporting as black increased sharply as the proportion of African ancestry reached about 20% in Salvador and 10% in Porto Alegre. The probability of self-reporting as white decreased sharply as the proportion of African ancestry reached about 10% in Porto Alegre, while in Salvador the curve decreased sharply from the start. Individuals who self-declared as brown showed a bell-shaped predicted probability of having African ancestry in all sites.

**Fig 3:**
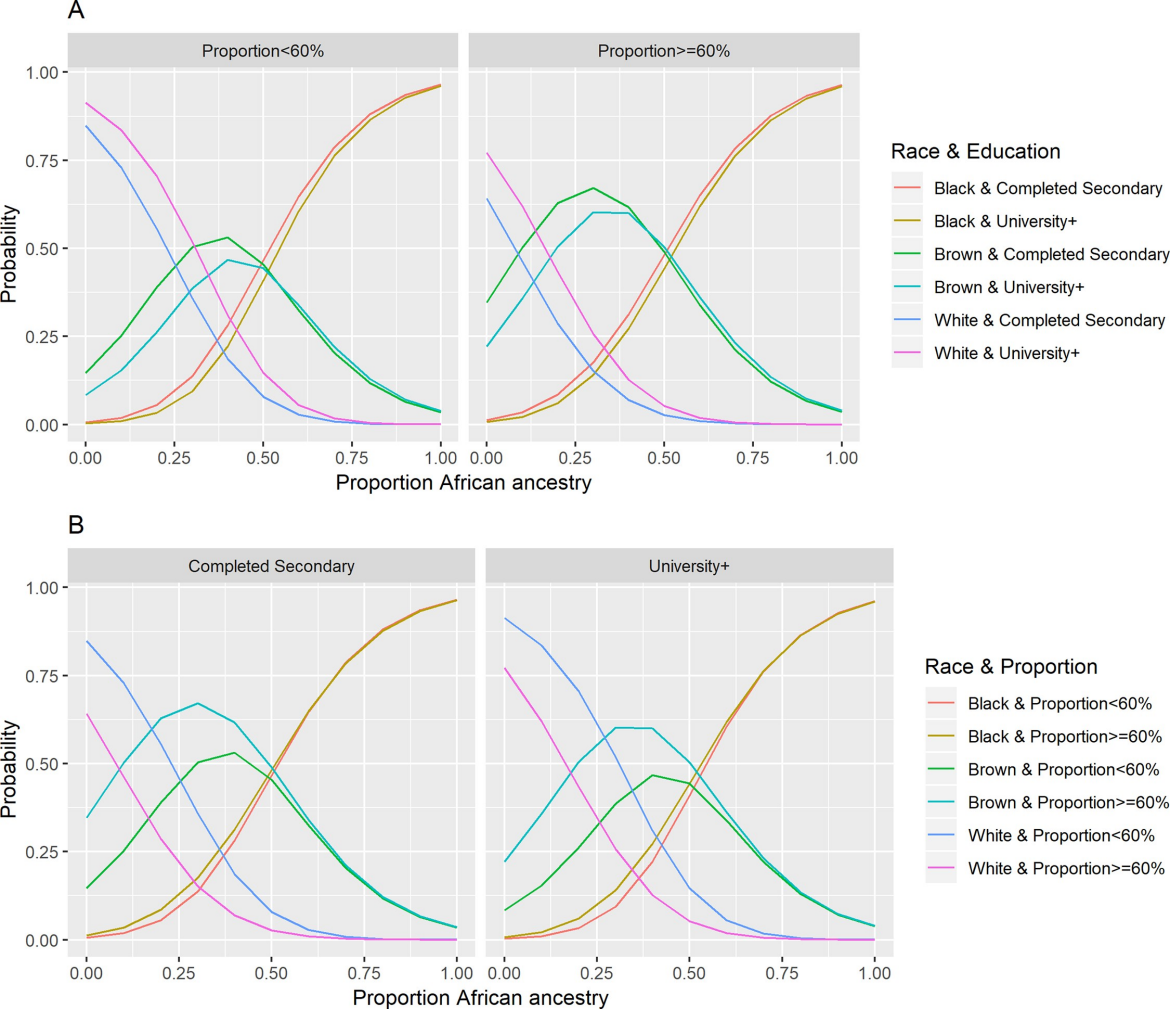
Predicted probability of ethnoracial self-classification as black, brown and white along the continuum of genomic proportion of African ancestry in all participants, and by (A) educational level and proportion of black and brown inhabitants (≥ 60% or < 60%) in the census tracts of residence (ELSA-Brasil) and (B) by proportion of black and brown inhabitants (≥ 60% or < 60%) in the census tracts of residence and by educational level (ELSA-Brasil).

Fig 3 shows the probability of self-classifying as white, black or brown according to African ancestry by educational level and racial composition of residential census tract (proportion of black and brown inhabitants ≥60% or <60%). The probabilities of self-declaring as black or brown with more than 50% of African ancestry showed the smallest differences between educational and racial composition strata. On the other hand, self-declaring as brown with up to 50% of African ancestry and as white with up to 75% of African ancestry were the ones with the greatest variation. Thus, for values of African ancestry proportion of up to 50%, the probability of self-declaring as brown was lower among participants with the higher educational level and also among those who resided in census tracts with the lower proportion of black and brown inhabitants. The probability of self-declaring as white decreased as the proportion of African ancestry increased, with differences among educational levels and the type of racial composition, up to values of 75%.

## Discussion

This study is possibly the largest investigation ever undertaken in Brazil regarding the relationships between race or colour classification and genomic ancestry profiles, given the number of participants, the sample’s geographic scope and the set of genomic markers. Results indicate that, as expected, there is a gradient of reduction of European genomic ancestry in the white, brown and black samples and, in turn, of increase of African ancestry. Our main results, in many aspects innovative, are as follows: 1. self-declaring as black or brown varies not only according to the proportion of African ancestry, but also according to the proportion of black and brown inhabitants in residential census tracts and according to the city where they are located; 2. racial composition of residential census tracts is not only associated with self-declared race but it interacts with ancestry so that different results are presented for strata of racial composition of individuals’ closest context; 3. holding educational level constant, the racial composition of the census tract is associated with the probability of self-identifying as white, and especially as brown, but not as black; 4. holding the racial composition of the census tract constant, the educational level is associated with the probability of self-classifying as white, and especially as brown, but not as black.

In general, our results resonate the findings of previous studies (see Lima-Costa et al. 2015[17]; Pena et al. 2011[19]) regarding patterns of associations between genomic ancestry and race-colour classification in Brazil. As expected, subjects classified as white present the highest levels of European genomic ancestry and those classified as black the lowest (and the inverse for African ancestry), at the same time that distribution patterns markedly vary across the regions of the country, reflecting the different histories of the formation of the Brazilian population along the past five centuries. Also in a similar fashion to reported in Lima-Costa et al. (2015)[17], our study points to a bell-shaped predicted African genomic ancestry in those subjects self-classified as brown. As for further comparisons, we were unable to identify studies carried out in Brazil which, based on samples from different regions of the country and with comparable methodology, have jointly evaluated not only the influences of individual socio-economic characteristics, but also supra-individual characteristics, such as the race/colour composition of participants’ areas of residence, as we performed in our study.

We recognize that our analyses refer to dimensions of race or colour (self-classification and biological ancestry) that are linked to different dimensions (socio-cultural and biological), though they may interface in ways that are reflected in subjects’ perceptions of their ethnic-racial belonging. While individuals, in their daily lives, are frequently questioned and/or experience the dimension of racial or colour belonging (white, black, brown, etc.), their biological ancestry profile, established through quantification according to genetic parameters, is not present in their everyday lives, unless they are exposed to specific lab tests. In choosing to set self-classification as the dependent variable in our statistical models, we do not intend to suggest that subjects’ perceptions of their belonging according to race or colour categories may be cognitively explained by their genomic profiles, of which, in principle, they are unaware. Despite the existence of a strong relationship between these planes, to the extent that previous studies carried out in Brazil and other Latin American countries [15–17,21,22] have shown that, when questioned about their perceptions of their biological ancestry, lighter-skinned individuals tend to declare having a greater European ancestry, while those with darker skin tend to declare having greater African ancestry. It is possible that these patterns of connection between self-classification and biological ancestry occur due to the relationship between ancestry markers and the expression of morphological traits, such as skin colour and other physical attributes, which are relevant to the perception of belonging to race or colour categories.

For many decades, analyses of the inter-relations between sociocultural and economic factors involved in the Brazilian race or colour classification have drawn the attention of social scientists, especially from the fields of Anthropology and Sociology. These studies, which were intensified from the 1950s onwards, have produced consistent evidence that shows that the classification of race or colour in Brazil is strongly influenced by cultural and socio-economic aspects [5–9,11–13], which has even led to the proposal of the “social race” concept [32]. Considering this context, surprisingly, recent genetic population research and epidemiological studies focusing on the investigation of the agreement between self-declared race and genomic ancestry have yet to take into account, in a systematic fashion, the influence of sociocultural and economic aspects in their analytical models [15–20,23].

Studies that sought to analyse the inter-relations between race or colour classification and genomic profiles have received growing attention in Brazil. However, with few exceptions, studies have been based on reduced and/or geographically limited samples and/or have grouped populations from different age groups in which data collection strategies regarding the race/colour information were not the same [15,17,19,21]. Of note, there are strong relations between socioeconomic conditions and the area of residence in Brazil, a country with high rates of inequality[33]. Although census tracts are logistic units related to the amount of properties and people, they present an internal homogeneity regarding level of education and income. These characteristics are powerful determinants of residential area, stronger than personal preferences.

Echoing findings from other studies carried out in Brazil [15–19, 21–23], our results indicate that there is a strong association between race/colour self-classification and biological ancestry, stronger in Porto Alegre and weaker in Salvador, and also that the highest agreement between self-classification and genomic ancestry occurs at the extremes of the predicted classification distribution in the case of black and white individuals. However, when considering socio-demographic characteristics, as we have in this study, this scenario becomes more complex, especially for white and brown individuals. This is due to the fact that, within the set of individuals with lower proportions of African ancestry, there is a large variation in the probability of self-classifying as brown and as white and, to a lesser extent, as black. One may assume that the higher proportion of African ancestry among individuals self-classified as black or brown (at the upper limit of the proportion of African ancestry) is the most plausible explanation for the lower influence of the educational level and of the racial composition of the residential census tract. It is also possible that the strong challenges to the racial democracy narrative, led by black activists, but that have spread to a large portion of the Brazilian population over the past two decades, may also explain the lower variability according to these characteristics [6,11,34]. The increase in the proportion of those who self-classify as black in the National Census, which recently happened for the first time since 1940, reinforces this hypothesis, since this group represented 5% of the population in 1991, 6% in 2000 and 8% in 2010. According to Miranda (2015) [35], this increase is primarily due to racial reclassification, and not to demographic events.

The greater propensity toward “whitening” in groups with higher educational level in Brazil, depending on different contexts and social circles, has been described in multiple socio-anthropological studies [5,6,9,10,13,14], though it remains under-investigated in studies more directly focused on analysing patterns of agreement between race or colour classification and genomic ancestry [21,22]. If this first dimension was already well-known and is reinforced by our results, we believe that the most innovative dimension of our research is the fact that we have shown that race or colour composition of individuals’ closest context–their place of residence–influences their race or colour self-classification. Therefore, biological ancestry is important, but not sufficient, when predicting self-declared race, in which different demographic, economic and social indicators relating to the territory, in addition to individual characteristics, interfere. Specifically, for the same proportion of African ancestry and the same educational level, we have found that the probability of self-classifying as brown increases, and that of self-identifying as white decreases, according to the increase in the proportion of black and brown inhabitants in the residential census tract.

Our results empirically confirm Muniz and Bastos’ (2017) [12] description of the many dimensions related to what they have called the “uncertainty in racial classification” in Brazil, emphasizing what they have called “contextual aspects”. Among these, regarding the dimension “space”, authors state that “areas with a high proportion of whites are expected to have individuals with greater potential to change their racial classification […], tending to classify themselves as whites. Meanwhile, in areas with a high proportion of *pretos* [black individuals], there might be a greater tendency for *pardos* [brown individuals] to classify themselves as black” (2017:5).

Inter-relations between space and the ethnic-racial issue have been widely studied in countries like the United States, among others, where patterns of residential segregation have been shown to be structuring, from a socio-economic standpoint, as well as with regard to health indicators [36]. In Brazil, the occurrence of residential segregation related to ethnic-racial belonging has been less studied than in other countries [9], but there is evidence that it is associated with the occurrence of chronic diseases [37]. Thus, even though specific mechanisms must still be better understood, this study brings an under-explored perspective to the consideration of the space dimension in terms of the social composition of the place of residence.

One characteristic of the Brazilian race/colour classification system, such as it is used in official records, is the existence, since the 1940s, of a category related to miscegenation: “brown” [8,9,35]. All national censuses carried out in Brazil, the first of which took place in 1872, have included a category related to “mixed race”, though there have been variations in terminology over time. It is worth noting that, over the course of Brazilian history, the category “brown” and its variations have both been associated with notions of “racial degeneration”, such as in the late 19^th^ and early 20^th^ centuries, when scientific racism predominated, and have been portrayed as a symbol of “Brazilianness”, starting in the 1930s, when the sociocultural and political dimensions of miscegenation started being valued. In recent decades, when debates surrounding racial relations and inequalities became increasingly influenced by multicultural perspectives, there has been an emphasis on the polarization of racial identities in Brazil, on the white-black axis, with a decrease in the number of people who identify as “brown” [6,35]. Another relevant aspect of the “brown” category concerns its multiplicity of connotations, depending on the region of the country, related to the different trajectories of colonization processes over the centuries. For example, if, in the Northern region, and in the Amazon in particular, the category “brown” is closely, though not exclusively, associated with the miscegenation between white and indigenous individuals, further south it is predominantly linked with the miscegenation between white and black individuals.

Reflecting the issues raised above, our results indicate that, when compared with the categories white and black, those who identify themselves as brown showed the greatest variability–estimated through the variation coefficient of the maximum values of predicted probabilities for each city and each self-declared race/colour (Fig 2). For example, if we take the proportion of African genomic ancestry of 0.25–0.30, the probability that an individual self-classified as brown was approximately 0.30 in Porto Alegre and around 0.60 in Salvador. The metropolitan regions where these two cities are located have contrasting profiles: the former with a higher proportion of white and lower proportion of brown and black inhabitants, due to the more intense European immigration over the centuries (82.6%, 7.6% and 9.3%, respectively, according to the 2010 Census); the latter, situated in one of the regions (North-East) that most received persons of African origin, as a result of slave trafficking, has a population that is 18% white, 27% black and 53% brown [27]. A suggestive pattern that emerges when comparing the samples from the many centres is that in areas where Afro-Brazilian populations predominate, based on the composition of black and brown inhabitants, the probability of a person self-declaring as brown at the 0.25–0.30 range of African ancestry is greater than in areas where white populations predominate. The opposite is also true, that is, in areas that are “whiter”, people with 0.25–0.30 African ancestry will tend to self-declare as brown less often.

Variations in race-colour composition observed in the various Brazilian regions might also help to explain the differential effect of the amount of African ancestry on self-declaring as black. For instance, our findings show that an increase of 10% in African ancestry increases the odds of self-identifying as black 14 times in Porto Alegre compared to 4 times in Salvador (Table 2). Muniz and Bastos (2017) [12] suggest that Brazilian towns whose populations mostly derive from European stocks, in particular those in the Southern part of the country might present “more rigid racial boundaries”. Compared to other parts of the country, major towns in Southern Brazil present much lower percentages of their populations composed by individuals who self-classify as brown (mestizo). It is possible that, in places where racial-colour composition is closer to the binary pattern (black and white), as it is the case of Porto Alegre, genomic ancestry and colour/race classification present particular patterns of association, differing from those prevailing in more admixed regions of the country.

The interpretation of the results presented herein should consider a few limitations. ELSA-Brasil is not a population-based study. Given the nature of the sample–civil servants from universities and research institutions–those at the extremes of the Brazilian income distribution (i.e. the highest- and the lowest-income strata individuals) are not represented. In addition, individuals (especially blacks and browns) from our sample have higher educational achievements in comparison to the general Brazilian population. Despite these characteristics of our study population, it would not be expected to affect association estimates. Another potential limitation is the use of Pima and Maya individuals as reference samples for the Native American continental ancestry component of our estimates. Nonetheless, although this can lead to less accurate estimates of this continental component, we have benchmarked our estimates using different combinations of the publicly available data for South American populations from both 1000G and HGDP projects with no observable significant difference regardless of the combination of reference samples used [30].

The race or colour classification is a complex and controversial topic in the scientific debate and in policy-making. Though it is defined in the Social Sciences as a social construct–a social conception with strong consequences for institutions and interpersonal relations–the growing discussion regarding genomics requires that we pay special attention to conceptual frameworks. If, on the one hand, self-declared race/colour is a social construct, genomic research is focused on ancestry, i.e., individuals’ genetic lineage. Since these are different concepts and answer different scientific questions, neither classification system can be considered as “the truth” regarding race. What each represents must be understood in light of the approaches and issues under investigation.

Our results indicate that ancestry is a strong predictor of self-declared race/colour. However, it cannot explain the set of factors that condition race/colour, which is influenced both by individual social characteristics and by the characteristics of the context in which people live. We should therefore be cautious regarding attempts to use only biological ancestry as a marker of race or colour. In the scientific field, when researchers attempt to “substitute” self-declared race for something regarded as more reliable (since it is biological), they lose access to socio-anthropological and subjective phenomena revealed by self-classification. Thus, a scientifically delicate and ethically damaging attitude is affirmed, since the entire trajectory of discrimination and socio-economic disadvantage of several generations of black and brown individuals is lost, as only self-declared race is a marker of these conditions.

## Supporting information

S1 Table Comparison between included and excluded participants according to selected characteristics. ELSA-Brasil, 2008–2010.
(DOCX)

S2 Table African, European and Amerindian proportion medians, by research centre and self-declared race/colour. ELSA-Brasil, 2008–2010.
(DOCX)

S3 Table Odds ratio of self-declaring as black (compared to white) according to research centre and total. ELSA-Brasil, 2008–2010 (using the proportion of black instead of black and brown inhabitants in the census tracts).
(DOCX)
