# Supplementary material for: Context-dependence of race self-classification: Results from a highly mixed and unequal middle-income country
Source: PLoS One. 2019 May 16;14(5):e0216653. doi: 10.1371/journal.pone.0216653 (PMC6522012; doi:10.1371/journal.pone.0216653)
Supplement: S1 Table — ELSA-Brasil, 2008–2010. (DOCX) [file pone.0216653.s001.docx]

| Variables | Excluded (%) | Included (%) | Total (%) | P-value |
| --- | --- | --- | --- | --- |
| **Total** | 5772 | 9333 | 15105 | - |
| **Colour/race** |  |  |  |  |
| Asian descents | 374 (6.7) | 0 (0) | 374 (2.5) | < 0.001* |
| Black | 902 (16.1) | 1495 (16) | 2397 (16.1) |  |
| Brown | 1456 (26.1) | 2746 (29.4) | 4202 (28.2) |  |
| White | 2699 (48.3) | 5092 (54.6) | 7791 (52.2) |  |
| Indigenous | 157 (2.8) | 0 (0) | 157 (1.1) |  |
| **Educational level** |  |  |  |  |
| Completed secondary | 2562 (44.4) | 4593 (49.2) | 7155 (47.4) | <0.001* |
| University or + | 3210(55.6) | 4740 (50.8) | 7950 (52.6) |  |
| **Income (USD)** |  |  |  |  |
| < 500.00 | 2029 (35.4) | 3532 (38) | 5561 (37) | < 0.001* |
| 501.00 to 1000.00 | 1922 (33.5) | 3221 (34.6) | 5143 (34.2) |  |
| >1000.00 | 1781 (31.1) | 2550 (27.4) | 4331 (28.8) |  |
| **Age** |  |  |  |  |
| median(IQR) | 53 (46-60) | 50 (45-57) | 51 (45-58) | < 0.001** |
| **Sex** |  |  |  |  |
| M | 2578 (44.7) | 4309 (46.2) | 6887 (45.6) | 0.074* |
| F | 3194 (55.3) | 5024 (53.8) | 8218 (54.4) |  |
| *Chisq test **Ranksum test |  |  |  |  |
