# Supplementary material for: Context-dependence of race self-classification: Results from a highly mixed and unequal middle-income country
Source: PLoS One. 2019 May 16;14(5):e0216653. doi: 10.1371/journal.pone.0216653 (PMC6522012; doi:10.1371/journal.pone.0216653)
Supplement: S2 Table — ELSA-Brasil, 2008–2010. (DOCX) [file pone.0216653.s002.docx]

| Genomic ancestry | Salvador | Vitoria | Belo Horizonte | Rio de Janeiro | São Paulo | Porto Alegre | Total |
| --- | --- | --- | --- | --- | --- | --- | --- |
| **African** |  |  |  |  |  |  |  |
| White | 9.0 | 4.3 | 8.9 | 8.9 | 9.2 | 4.0 | 8.3 |
| Brown | 32.7 | 21.4 | 23.1 | 23.0 | 29.4 | 20.2 | 26.4 |
| Black | 63.5 | 49.9 | 58.8 | 52.3 | 52.5 | 52.2 | 56.4 |
| **European** |  |  |  |  |  |  |  |
| White | 83.5 | 87.3 | 84.1 | 80.5 | 79.0 | 86.5 | 81.8 |
| Brown | 58.1 | 64.0 | 67.9 | 58.6 | 53.8 | 56.7 | 59.0 |
| Black | 28.7 | 36.0 | 35.2 | 30.3 | 28.4 | 33.3 | 30.1 |
| **Amerindian** |  |  |  |  |  |  |  |
| White | 7.7 | 7.2 | 6.3 | 10.9 | 11.0 | 8.8 | 9.4 |
| Brown | 7.4 | 12.2 | 7.7 | 14.9 | 15.0 | 17.0 | 11.4 |
| Black | 5.9 | 9.8 | 5.0 | 10.7 | 11.4 | 12.7 | 9.0 |
