# Supplementary material for: Context-dependence of race self-classification: Results from a highly mixed and unequal middle-income country
Source: PLoS One. 2019 May 16;14(5):e0216653. doi: 10.1371/journal.pone.0216653 (PMC6522012; doi:10.1371/journal.pone.0216653)
Supplement: S3 Table — ELSA-Brasil, 2008–2010 (using the proportion of black instead of black and brown inhabitants in the census tracts). (DOCX) [file pone.0216653.s003.docx]

| Variables | Salvador | Vitória | Belo Horizonte | Rio de Janeiro | São Paulo | Porto Alegre | Overall |
| --- | --- | --- | --- | --- | --- | --- | --- |
|  | OR# (CI 95%) | OR# (CI 95%) | OR# (CI 95%) | OR# (CI 95%) | OR# (CI 95%) | OR# (CI 95%) | OR# (CI 95%) |
|  |  |  |  |  |  |  |  |
| **African ancestry (10%)** | 4.01 (2.99-5.39) | 9.21 (4.24-19.99) | 9.13 (5.84-14.25) | 4.47 (3.54-5.65) | 2.84 (2.61-3.09) | 14.12 (6.07-32.84) | 3.47 (3.22-3.73) |
| **Educational level** |  |  |  |  |  |  |  |
| (Ref = completed secondary) | 1.00 | 1.00 | 1.00 | 1.00 | 1.00 | 1.00 | 1.00 |
| University or + | 0.26 (0.1-0.65) | 0.88 (0.2-3.94) | 0.39 (0.16-0.99) | 0.63 (0.31-1.25) | 0.41 (0.29-0.58) | 1.05 (0.12-8.99) | 0.42 (0.32-0.55) |
| **Income (USD)** |  |  |  |  |  |  |  |
| (Ref = <500.00) | 1.00 | 1.00 | 1.00 | 1.00 | 1.00 | 1.00 | 1.00 |
| 501.00 to 1000.00 | 0.46 (0.16-1.31) | 0.57 (0.12-2.75) | 1.55 (0.56-4.26) | 0.17 (0.08-0.38) | 0.62 (0.45-0.86) | 0.49 (0.07-3.52) | 0.56 (0.43-0.72) |
| >1000.00 | 0.93 (0.34-2.55) | 0.21 (0.03-1.73) | 1.44 (0.39-5.27) | 0.17 (0.07-0.41) | 0.55 (0.35-0.87) | 1.06 (0.1-10.95) | 0.55 (0.39-0.76) |
|  |  |  |  |  |  |  |  |
| **Age (10 years)** | 0.6 (0.38-0.95) | 1.06 (0.47-2.4) | 0.86 (0.54-1.37) | 1.08 (0.75-1.55) | 1.04 (0.88-1.24) | 1.09 (0.46-2.56) | 0.96 (0.84-1.09) |
| **Sex** |  |  |  |  |  |  |  |
| (Ref = Male) | 1.00 | 1.00 | 1.00 | 1.00 | 1.00 | 1.00 | 1.00 |
| Female | 0.95 (0.42-2.14) | 0.89 (0.26-3.1) | 0.76 (0.33-1.76) | 2.65 (1.46-4.81) | 1.09 (0.83-1.44) | 1.04 (0.22-4.9) | 1.19 (0.96-1.47) |
|  |  |  |  |  |  |  |  |
| **Proportion of black inhabitants (10%)*** | 1.2 (0.81-1.76) | 1.23 (0.22-6.85) | 1.13 (0.48-2.69) | 2.07 (1.23-3.48) | 2.53 (1.82-3.51) | 1.48 (0.44-5.04) | 1.82 (1.5-2.21) |
| # mutually adjusted * by census tracts |  |  |  |  |  |  |  |
